# Pyroptosis- and Necroptosis-Related Signaling in Salicylate UV Absorber-Induced Toxicity: Implications for Sustainable Chemistry and Human Health

**DOI:** 10.3390/ijms27114777

**Published:** 2026-05-26

**Authors:** Chunlu He, Yan Wang, Jialiang Lin, Zihao Yu, Yuan Shi, Jianhua Cheng, Yunyun Jiang, Litao Hu

**Affiliations:** 1 The Key Laboratory of Pollution Control and Ecosystem Restoration in Industrial Clusters, The Ministry of Education, College of Environment and Energy, South China University of Technology, Guangzhou 510006, China; 2 South China Institute of Collaborative Innovation, Dongguan 523808, China

**Keywords:** 2-ethylhexyl salicylate, homosalate, mouse embryonic fibroblasts 3T6 cells, mitochondrial damage, DNA damage, zebrafish

## Abstract

As emerging global environmental contaminants, organic ultraviolet absorbers (OUVAs) are widely used in personal care formulations and exhibit environmental persistence and potential bioaccumulation. Among these compounds, 2-ethylhexyl salicylate (EHS) and homosalate (HMS) are the most frequently used salicylate-type UV filters in cosmetic formulations. Although an increasing number of studies have demonstrated their environmental hazards, little is known about the molecular mechanisms underlying their cytotoxicity in mammalian systems, a fundamental knowledge gap for both human health protection and the development of more environmentally friendly consumer goods. In this study, we used mouse embryonic fibroblasts (MEFs, 3T6) and zebrafish as models to assess the toxicological phenotypes of EHS and HMS in vitro and in vivo, respectively. We found that both EHS and HMS induced cellular damage characterized by oxidative stress, disrupted intracellular calcium homeostasis, mitochondrial impairment, and DNA damage. Importantly, molecular analyses further suggested the concurrent activation of two distinct regulated cell death programs: pyroptosis, as suggested by *Caspase-11*-mediated GSDMD cleavage, and necroptosis, as suggested by *ZBP1-RIPK3-Caspase-8*-mediated MLKL phosphorylation. The in vitro data have been partially validated at the level of gene expression and in developmental toxicity in the zebrafish model, providing some in vivo phenotypic and molecular correlates. While the upstream events were experimentally verified, the causal links among them remain to be further elucidated. Taken together, this work suggested that OUVA-induced toxicity is not limited to isolated oxidative damage, but may also involve the activation of two different cell death programs. These findings provide important molecular clues to understanding the potential health and ecological risks of widely used UV filters and offer a scientific basis for their more environmentally friendly safety evaluation and regulatory management, which are crucial for advancing more sustainable chemistry and safer consumer goods.

## 1. Introduction

Moderate ultraviolet (UV) radiation is beneficial for human health because it promotes vitamin D synthesis [1]. However, it is also a well-established inducing factor of skin damage and carcinogenesis [2]. To mitigate such damages, organic ultraviolet absorbers (OUVAs), as a class of highly conjugated organic compounds with the ability to effectively absorb UV radiation, have become essential ingredients widely used in personal care products such as sunscreens [3]. Especially, salicylate derivatives, including 2-ethylhexyl salicylate (EHS) and homosalate (HMS), are mostly used due to their broad-spectrum (UVA/UVB) absorption property [4].

Due to their extensive use and environmental persistence, OUVAs have been reported in rivers, lakes, and oceans [5,6,7,8,9,10]. More concerning is their potential for bioaccumulation; these compounds have been detected in human biological matrices, including blood, urine, and breast milk, and therefore are likely to enter the human body through a direct route of exposure [11,12,13]. For instance, the median concentration of EHS in indoor air particulate matter was reported to be 28.55 ng/m^3^ [14], and EHS at an environmentally relevant concentration (7.46 μg/L) was reported to induce reproductive toxicity in marine copepods [15]. The internal exposure of EHS in the German population was evaluated by monitoring the levels of their specific metabolites, 5cx-EPS, in urine. The levels of 5cx-EPS increased significantly between 1996 and 2020, and reached 0.06 μg/L in 2020 [16]; similarly, the concentrations of HMS metabolites were quantified in human urine at >0.02 μg/L [17]. Despite the clear evidence of ecological endocrine disruption, including causing coral bleaching and developmental toxicity in zebrafish [18,19,20,21], the molecular mechanisms responsible for the cytotoxicity of these compounds in mammalian cells remain largely elusive.

So far, apical endpoints like oxidative stress, endocrine disruption, or non-specific cytotoxicity in different cell models are the focus of current toxicological investigations of OUVAs [22,23,24]. However, there is still a great gap in knowledge regarding the specific signaling networks that mediate cell fate after OUVA-induced injury. Especially, little is known about whether, and how, these chemicals may interact with regulated cell death pathways, which are crucial for toxicity and disease. For example, programmed cell death modalities, such as pyroptosis and necroptosis, are rigorously regulated by specific molecular cascades (e.g., the Caspase-11-GSDMD and RIPK1-RIPK3 axis) and serve critical functions in stress responses, inflammation, and tissue homeostasis. Their activation by environmental pollutants represents a highly sophisticated, yet enigmatic level of toxicology.

Given the widespread presence of OUVAs in aquatic environments and human biological matrices, we face a major sustainability challenge. As persistent and potentially bioaccumulative chemicals, UV filters represent an unintended environmental and health impact of consumer product design [25,26]. Within the context of the United Nations Sustainable Development Goals (SDGs), particularly SDG 3 (Good Health and Well-being), SDG 6 (Clean Water and Sanitation), and SDG 12 (Responsible Consumption and Production), there is an increasing demand to assess and mitigate the impact of emerging contaminants [27]. To date, regulatory efforts targeting OUVAs have predominantly focused on ecological endpoints in aquatic organisms. However, our study suggests that these compounds can interfere with fundamental cellular processes in mammals and, in zebrafish embryos, also in vivo. Thus, the present work provides an essential piece of the puzzle to define the sustainability quotient of these personal care products, namely their human health risk, by unraveling the molecular mechanisms underlying OUVA-induced cytotoxicity. It is, therefore, not only a prerequisite for developing safer alternatives, guiding regulatory policymaking, and promoting the circular economy through sustainable chemistry, but also an essential step towards the definition of truly sustainable personal care products that protect both ecosystem and human health.

For these reasons, we used mouse embryonic fibroblasts (3T6, a sensitive in vitro model) and zebrafish embryos as an in vivo evaluation model to investigate the cytotoxic mechanisms of two widely used salicylate OUVAs, EHS and HMS. According to the permissible concentration standards of organic UV sunscreen agents and preliminary toxicity screening experiments, the exposure concentration is chosen within the range where the survival rate is 50–90% [28,29,30], to facilitate the mechanistic investigation of cell death pathways.

We tested the hypothesis that OUVAs induce a coordinated cellular stress response leading to programmed cell death. We found that EHS and HMS trigger a series of cellular damage responses that include oxidative stress, Ca^2+^ dyshomeostasis, mitochondrial dysfunction, and DNA damage. Whereas previous studies have largely focused on oxidative stress and apoptosis as toxic endpoints of UV filters, the possibility that pyroptosis and necroptosis are concurrently activated has not been explored. This study, including the upregulation of pathway-specific genes and the detection of cleaved GSDMD and phosphorylated MLKL, provides initial evidence suggesting that these salicylate-based sunscreens may simultaneously engage both pyroptosis and necroptosis as potential execution mechanisms. These findings point toward a model in which OUVA toxicity extends beyond generalized oxidative damage to an orchestrated cell death signaling network, thereby offering a mechanistic basis for the health risk assessment of these ubiquitous environmental contaminants.

## 2. Results

### 2.1. EHS and HMS Suppress the Proliferation of Mouse Embryonic Fibroblasts

**Figure 1:**
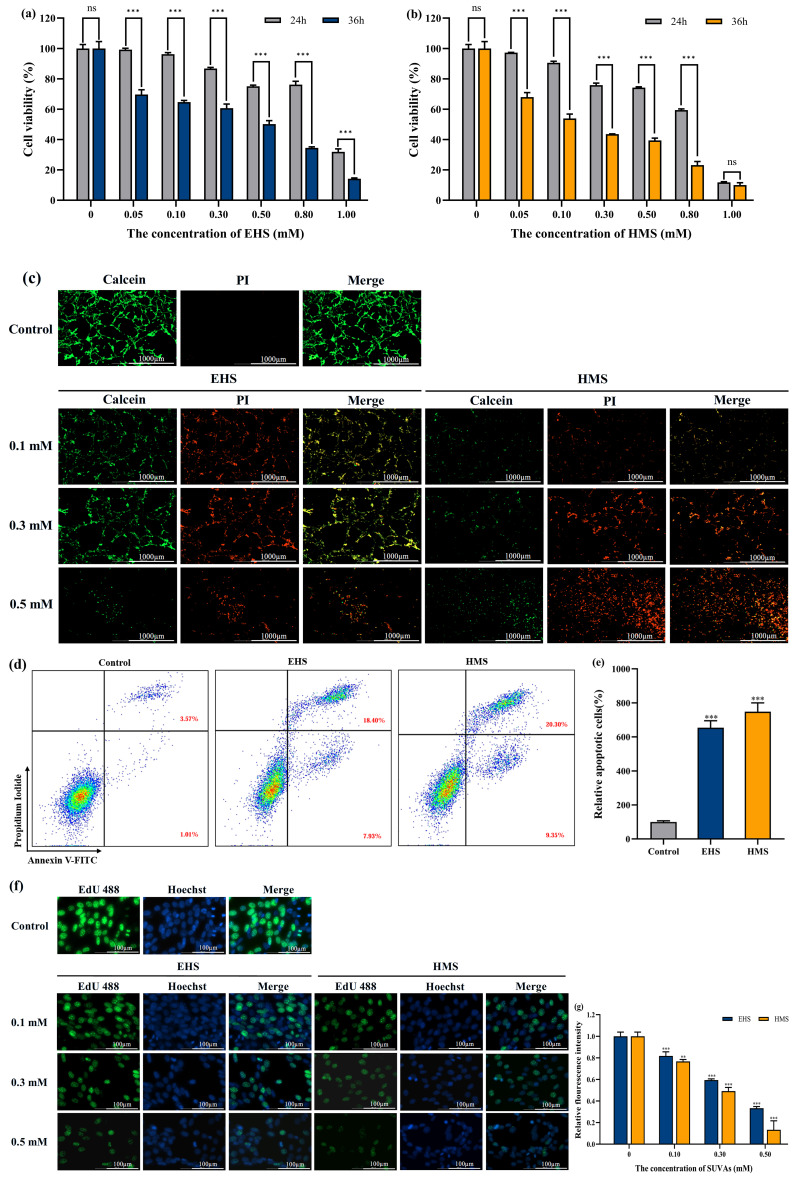
EHS and HMS inhibit 3T6 cell proliferation. (**a**,**b**) Viability assessed by CCK-8 after 24 and 36 h exposure to 0.05–1.00 mM EHS (**a**) or HMS (**b**). (**c**) Live/Dead staining after 36 h exposure to 0.10, 0.30, 0.50 mM EHS or HMS. (**d**,**e**) Flow cytometry analysis of apoptosis (Annexin V-FITC/PI) after 36 h exposure to 0.30 mM EHS or HMS: representative dot plots (**d**) and quantification of early and late apoptotic cells (**e**). (**f**,**g**) EdU incorporation assay after 36 h exposure to EHS or HMS (0.10, 0.30, 0.50 mM): representative images (**f**) and quantification of EdU-positive cells (**g**). Data are mean ± SD (*n* = 3) and analyzed by one-way ANOVA with Tukey’s test. ** *p* < 0.01,*** *p* < 0.001.

Cell viability is a commonly used metric for evaluating the toxicity of exogenous substances [31]. In order to preliminarily understand the effects of EHS and HMS on 3T6 cells, we exposed the cells to six different concentrations of each compound, all well below the maximum levels allowed under current regulations. According to China’s Safety and Technical Standards for Cosmetics (2015 edition) and relevant EU regulations [32], the maximum permitted concentration is 5% (approximately 0.20 mol/L) for EHS and 10% (approximately 0.38 mol/L) for HMS. As shown in Figure 1a,b, exposure to either sunscreen ingredient led to a time- and concentration-dependent drop in cell viability. Even at the lowest concentration (0.05 mM) of both compounds, cells showed a significant reduction in viability at 36 h as compared to the control (EHS: 69.7% and HMS: 68.0% < 80%). The results suggested that HMS was slightly more toxic than EHS, because it reduced viability to a greater extent than EHS at the lowest concentration. Based on these findings, we chose three concentrations (0.10, 0.30, and 0.50 mM) for 36 h treatment in the subsequent experiments.

The results were also confirmed by live/dead staining (Figure 1c). With an increase in the concentration of EHS or HMS, the green fluorescence of viable cells was gradually weakened, and the red fluorescence of dead cells was gradually enhanced, which was a sign of cell death. The results of flow cytometry analysis were also consistent with the above (Figure 1d,e).

Apoptosis and proliferation are two opposing processes in the regulation of cell fate, and their balance is important for the maintenance of normal tissue homeostasis. To see whether EHS and HMS might also interfere with cell proliferation, we carried out an EdU-488 incorporation assay. As illustrated in Figure 1f,g, both compounds markedly reduced the number of EdU-positive (proliferating) cells compared with controls, suggesting an inhibitory effect on cell proliferation.

### 2.2. Exposure to EHS and HMS Increases ROS Levels in Embryonic Fibroblasts

Reactive oxygen species (ROS) are considered metabolic by-products of normal cell metabolism and have an important role in the maintenance of homeostasis. Normally, low levels of ROS promote cell proliferation and differentiation, whereas high levels of ROS induce oxidative stress, causing injury and apoptosis in cells [33].

**Figure 2:**
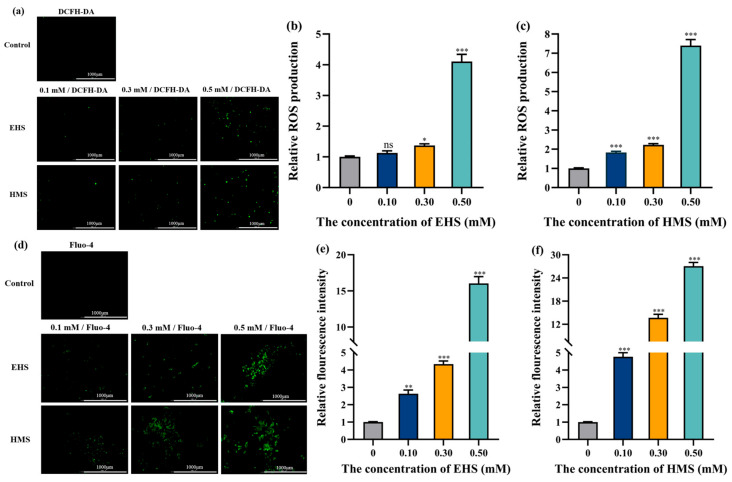
Intracellular ROS and Ca^2+^ levels in 3T6 cells after 36 h exposure to EHS or HMS (0.10, 0.30, 0.50 mM). (**a**–**c**) ROS measurement using DCFH-DA probe: representative images (**a**) and quantification for EHS (**b**) and HMS (**c**). (**d**–**f**) Ca^2+^ detection using Fluo-4 AM: representative images (**d**) and quantification for EHS (**e**) and HMS (**f**). Data are mean ± SD (*n* = 3). One-way ANOVA with Tukey’s test. * *p* < 0.05, ** *p* < 0.01, *** *p* < 0.001.

Noticing the reduction in cell viability, we next wondered whether changes in ROS levels common in cytotoxic phenotypes may also be the cause of cytotoxicity caused by EHS and HMS. We therefore investigated whether these two compounds could induce the production of ROS in 3T6 cells. As shown in Figure 2a, the intracellular ROS level (monitored using the DCFH-DA probe) increased significantly after exposure to the two compounds. The green fluorescence intensity was concentration-dependent (Figure 2a–c). These results suggested that both EHS and HMS can induce ROS accumulation in 3T6 cells. Importantly, when compared with EHS, HMS tested at the same concentration resulted in approximately 1.6- to 1.8-fold greater ROS production, suggesting that HMS induced much stronger oxidative stress in 3T6 cells.

We suspect that EHS and HMS act as pro-oxidants in 3T6 cells and induce the overproduction of other reactive molecules. The cellular antioxidant enzymes could partially remove these molecules, but their net effect was still oxidative stress, because the cellular antioxidant enzymes could not fully prevent the excess influx of ROS.

**Figure A1:**
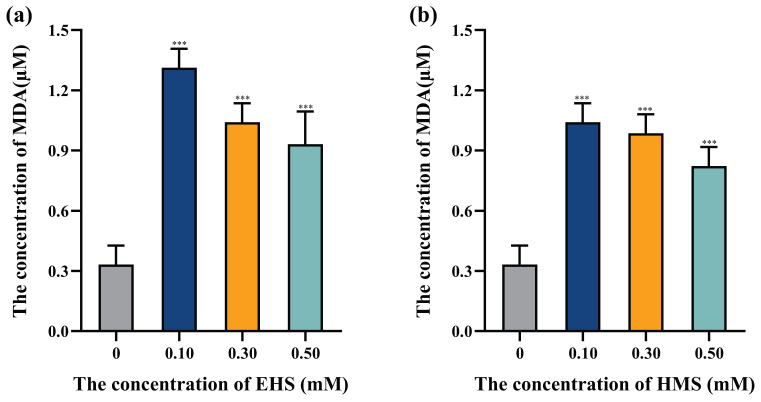
The change in MDA content of 3T6 cells at different SUVA concentrations after 36 h. (**a**): EHS, (**b**): HMS. *** *p* < 0.001.

**Figure A2:**
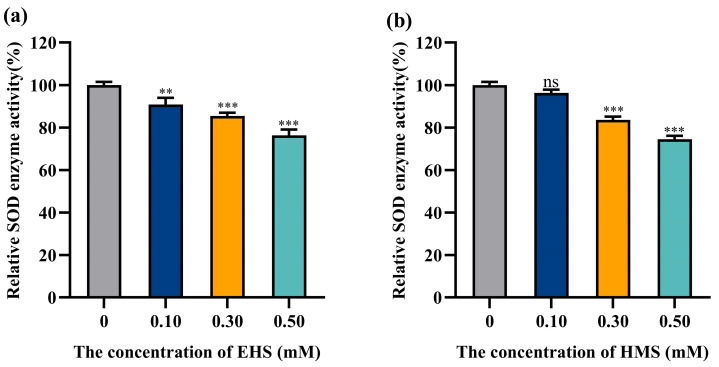
The change in relative SOD enzyme activity of 3T6 cells after different concentrations of SUVA for 36 h. (**a**): EHS, (**b**): HMS. ** *p* < 0.01, *** *p* < 0.001.

At the same time, we also further explored other indicators related to the cell’s oxidative stress, and the results showed that the cellular MDA content increased significantly (Figure A1), and SOD enzyme activity decreased significantly (Figure A2), suggesting that under EHS and HMS stimulation, the cell membranes had undergone oxidative damage, and the cells were in a state of oxidative stress.

### 2.3. Intracellular Ca^2+^ Homeostatic Imbalance After Exposure to EHS and HMS

It is well known that the maintenance of normal intracellular calcium levels is important for normal cell function; calcium ions play important roles in numerous physiological processes, from cell survival to cell death [34,35]. However, a large amount of ROS can disturb Ca^2+^ signaling [36]. Therefore, we next explored whether the oxidative stress induced by EHS and HMS could also disturb calcium homeostasis. Using Fluo-4 AM as a calcium indicator, we observed intracellular Ca^2+^ increased after treatment with either compound (Figure 2d). The green fluorescence signal intensified progressively with higher concentrations of EHS or HMS, pointing to a dose-dependent rise in Ca^2+^ levels. And, at the same concentrations, HMS consistently elicited a stronger response than EHS, suggesting it leads to a more pronounced disturbance of calcium homeostasis (Figure 2e,f).

### 2.4. EHS and HMS Induce Mitochondrial Damage

Given the well-established interplay between ROS and Ca^2+^ signaling, and their combined influence on mitochondrial function [37,38], we next explored how the elevated ROS levels and disrupted calcium homeostasis we observed might affect mitochondria. It is known that excessive ROS can oxidatively inactivate MICU1, a regulatory subunit of the mitochondrial calcium uniporter (MCU), leading to calcium overload within the mitochondria [39]. Conversely, an accumulation of Ca^2+^ in the mitochondrial matrix can overdrive the tricarboxylic acid cycle, further promoting ROS leakage from the electron transport chain; together, these events can trigger opening of the mitochondrial permeability transition pore [40,41]. With this in mind, we assessed several indicators of mitochondrial function, including membrane potential, ATP production, and mitochondrial superoxide levels, to better understand how ROS and calcium imbalance contribute to downstream mitochondrial damage.

**Figure 3:**
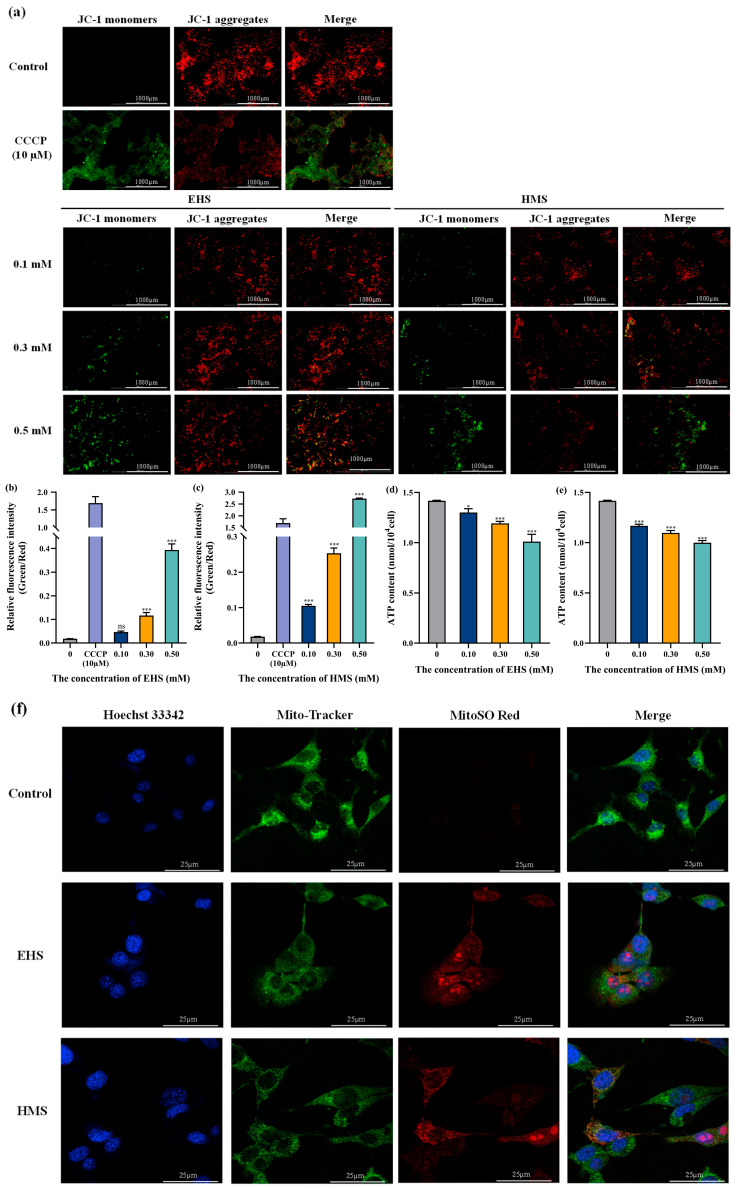
Mitochondrial damage in 3T6 cells after 36 h exposure to EHS or HMS (0.10, 0.30, 0.50 mM). (**a**) JC-1 staining images of mitochondrial membrane potential (MMP), with CCCP as a positive control. (**b**,**c**) Green/Red fluorescence ratio after EHS (**b**) or HMS (**c**) treatment. (**d**,**e**) Cellular ATP levels after EHS (**d**) or HMS (**e**) treatment. (**f**) MitoSOX Red staining for mitochondrial superoxide (Hoechst 33342: blue, nuclei; MitoTracker Green: green, mitochondria). Data are mean ± SD (*n* = 3). One-way ANOVA with Tukey’s test. * *p* < 0.05, *** *p* < 0.001.

A drop in mitochondrial membrane potential is an early characteristic marker of apoptosis, an irreversible sign of apoptosis [42,43]. Using JC-1 as a fluorescent probe, we examined MMP changes following EHS or HMS exposure. As demonstrated in Figure 3a, the control cells displayed bright red JC-1 fluorescence and little green signal, suggesting a high membrane potential. In contrast, the cells treated with 10 µM CCCP (positive control) almost lost the red fluorescence and displayed intense green staining. However, when the cells were treated with increasing concentrations of either EHS or HMS, a gradual change in fluorescence from red to green was observed, suggesting a dose-dependent dissipation of MMP. Notably, the green/red fluorescence ratio (Figure 3b,c) increased more markedly in HMS-treated cells than in those exposed to EHS, suggesting that HMS has a stronger impact on mitochondrial membrane integrity.

Because MMP serves as the main driving force for oxidative phosphorylation, and thus for ATP synthesis [44], we next measured cellular ATP levels after confirming MMP loss. As illustrated in Figure 3d,e, ATP content dropped significantly in a concentration-dependent manner following treatment with either compound, with HMS causing a more severe decrease than EHS at the same concentration.

Given the close source-sink relationship between overall intracellular ROS and mitochondrial superoxide, we also investigated where the observed ROS elevation originates. Mitochondria are known to be the primary site of intracellular ROS production; under normal conditions, roughly 0.25–11% of consumed oxygen is converted to mitochondrial superoxide [45]. More importantly, mitochondrial ROS can amplify the signal through a process known as “ROS-induced ROS release,” spreading oxidative stress to neighboring mitochondria and even to cytosolic redox systems, ultimately causing a surge in overall ROS levels [40]. This highlights the significance of differentiating between general intracellular ROS and mitochondrion-specific superoxide when analyzing oxidative damage pathways [46,47]. Having noted a definite increase in total ROS, we used MitoSOX™ Red, a fluorescent probe made for specifically detecting mitochondrial superoxide, to find the subcellular source of ROS and assess its direct relation to downstream mitochondrial dysfunction.

In healthy cells, MitoSOX™ Red mostly stays non-fluorescent. Upon reaction with superoxide, however, the probe redistributes and binds to both mitochondrial and nuclear DNA, producing a red signal in both compartments [48]. As shown in Figure 3f, exposure to EHS or HMS led to a marked increase in red fluorescence, suggesting an increase in mitochondrial superoxide levels.

### 2.5. EHS and HMS Cause Cellular DNA Damage

In our earlier EdU-488 experiments, we noticed that after Hoechst staining, some nuclei showed signs of shrinkage, marginalization, rupture, and uneven chromatin distribution (Figure 1f). This prompted us to consider whether exposure to EHS and HMS might also induce DNA damage. To explore that possibility, we carried out a set of experiments aimed at directly assessing the extent of DNA damage.

**Figure 4:**
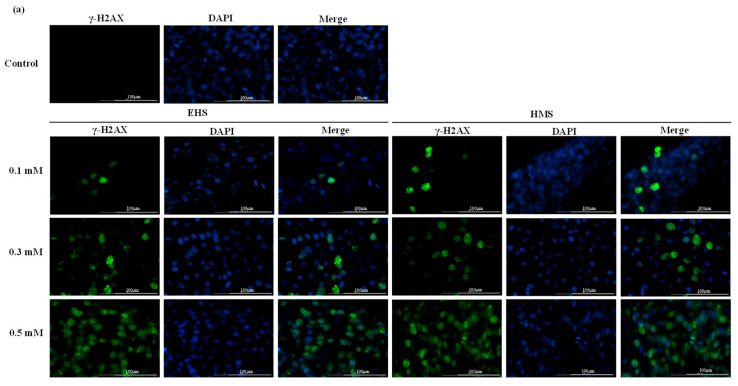

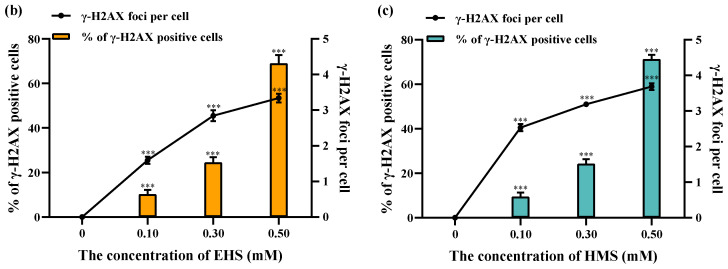
DNA damage in 3T6 cells exposed to EHS or HMS for 36 h. (**a**) Representative immunofluorescence images of γ-H2AX (green) and nuclei (blue) at concentrations of 0.10, 0.30, and 0.50 mM. (**b**,**c**) Quantification of γ-H2AX: percentage of positive cells and average number of foci per positive cell after EHS (**b**) or HMS (**c**) treatment. Data are mean ± standard deviation (*n* = 3). One-way analysis of variance (ANOVA) followed by Tukey’s test. *** *p* < 0.001.

Among various forms of DNA lesions, double-strand breaks (DSBs) are considered one of the most severe [49]. A widely used indicator of DSBs, and a key component of the DNA damage response, is the phosphorylation of H2AX, yielding γ-H2AX [50]. As shown in Figure 4a, increasing the concentration of EHS or HMS led to an increase in green foci and a stepwise enhancement of fluorescence intensity, reflecting elevated levels of γ-H2AX, suggesting more extensive DNA damage. Quantitative analysis further supported (Figure 4b,c) that both the proportion of positive cells and the average number of foci per positive cell increased in a concentration-dependent manner.

Taken together, these results suggest that both EHS and HMS are capable of causing genotoxicity, primarily through the induction of DNA double-strand breaks.

### 2.6. EHS and HMS Affect the Expression of Cell Death-Related Genes

Mitochondrial dysfunction and DNA damage are not only direct markers of energy failure and genetic damage, but also can be upstream events that trigger nuclear transcription programs. Given these considerations, we subsequently investigated how cells exposed to EHS or HMS modulate their gene expression profile, in an attempt to provide further insights into the toxicological mechanisms underlying these effects.

**Figure 5:**
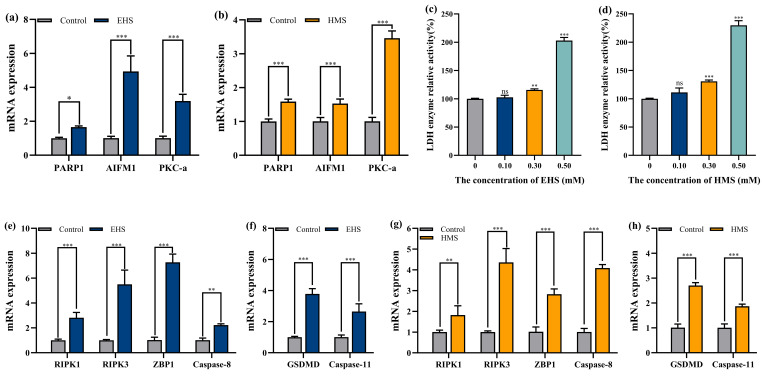
Gene expression changes and LDH release in 3T6 cells after 36 h exposure to 0.30 mM EHS or HMS. (**a**,**b**) mRNA levels of *PARP1*, *AIFM1*, and *PKC-α* after EHS (**a**) or HMS (**b**) exposure. (**c**,**d**) LDH release after EHS (**c**) or HMS (**d**) treatment. (**e**,**g**) mRNA levels of *RIPK1*, *RIPK3*, *ZBP1*, and *Caspase-8* after EHS (**e**) or HMS (**g**) exposure. (**f**,**h**) mRNA levels of *GSDMD* and *Caspase-11* after EHS (**f**) or HMS (**h**) exposure. Data are mean ± SD (*n* = 3). One-way ANOVA followed by Tukey’s test. * *p* < 0.05, ** *p* < 0.01, *** *p* < 0.001.

**Figure A3:**
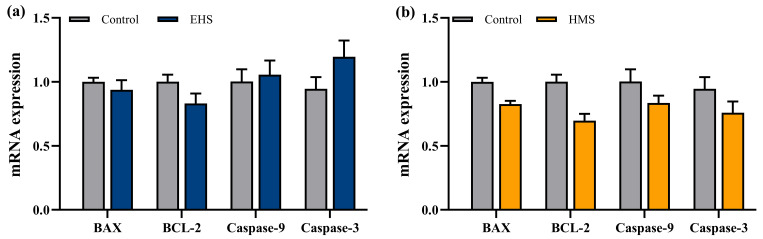
The change in gene expression of 3T6 cells after different concentrations of SUVA for 36 h. (**a**): EHS, (**b**): HMS.

Based on the results obtained above, we initially analyzed the expression of genes involved in mitochondrial function (*AIFM1*, *BAX*, *BCL-2*, *Caspase-3*, *Caspase-9*), DNA damage (*PARP1*), and calcium homeostasis (*PKC-α*). The results showed that *PARP1* and *PKC-α* were significantly upregulated in both cases and could therefore be considered to be highly correlated with our previous phenotypic results (Figure 5a,b). In contrast, no significant changes were observed in the expression of genes involved in the mitochondrial apoptosis pathway (Figure A3), suggesting that EHS and HMS did not induce cell death through the classic mitochondrial pathway.

Since mitochondria are located at the crossroads of apoptosis, pyroptosis, and necroptosis, damage to mitochondria, coupled with DNA injury, can drive cells toward different death phenotypes through pyroptotic or necrotic factors [51,52,53]. To systematically elucidate the types of cell death induced by EHS or HMS and the associated signaling pathways, we used the LDH release assay to evaluate cell membrane integrity. At the same time, we also analyzed the expression of key genes associated with forms of death related to membrane damage, such as pyroptosis-related molecules (*GSDMD*, *Caspase-11*) and necroptosis-related molecules (*RIPK1*, *RIPK3*, *ZBP1*, *Caspase-8*), to more clearly define the final execution pathway.

As shown in Figure 5c,d, the LDH release was significantly enhanced in a concentration-dependent manner following exposure to either of the two sunscreen agents, suggesting damage to the cell membrane. LDH release is a well-established marker of cytotoxicity, particularly necrotic cell death [54], and our results suggested that necroptosis may occur in this reaction.

Next, we analyzed the gene expression profiles of necroptosis and pyroptosis, and the results showed that EHS and HMS treatments significantly upregulated the expression of *GSDMD*, *Caspase-11*, *RIPK1*, *RIPK3*, *ZBP1*, and *Caspase-8* (Figure 5e–h).

**Figure 6:**
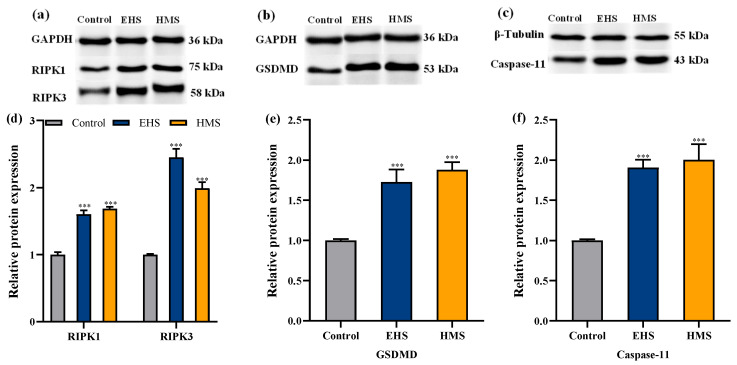

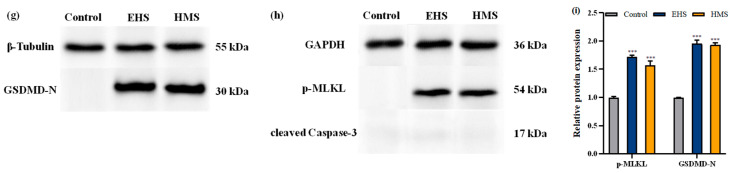
Cell death-related protein expression in 3T6 cells after 36 h exposure to 0.30 mM EHS or HMS. (**a**–**c**) Representative immunoblots of RIPK1/RIPK3 (**a**), GSDMD (**b**), and Caspase-11 (**c**). (**d**–**f**) Densitometric quantification of (**a**–**c**): (**d**) RIPK1 and RIPK3, (**e**) GSDMD, (**f**) Caspase-11. (**g**) Western blot of GSDMD-N. (**h**) Western blot of p-MLKL and cleaved caspase-3. (**i**) Quantification of (**g**,**h**). GAPDH and β-tubulin served as loading controls. Data are mean ± SD (*n* = 3); one-way ANOVA followed by Tukey’s test. *** *p* < 0.001.

We next analyzed the changes in protein expression by Western blot (Figure 6). EHS and HMS changed the expression of several proteins. The results of the loading controls (GAPDH and β-Tubulin) were consistent with those of the other samples, suggesting that the protein loading was consistent. Importantly, levels of RIPK1, RIPK3, GSDMD, and Caspase-11 were all significantly elevated in cells treated with either compound, further supporting the reliability of high mRNA gene expression.

Furthermore, to further verify pathway activation, we performed Western blot analysis for the cleaved GSDMD N-terminal fragment (GSDMD-N), phosphorylated MLKL (p-MLKL), and cleaved caspase-3. As shown in Figure 6g–i, both GSDMD-N and p-MLKL were detected at high levels in the EHS and HMS treatment groups, whereas no corresponding proteins were detected in the untreated control group. In contrast, cleaved caspase-3 was not detected in either the control or the treatment groups. These results are consistent with the activation of pyroptosis and necroptosis pathways and the absence of classical mitochondrial apoptosis pathway activation.

### 2.7. Rescue Experiments Support the Role of Mitochondrial Damage and DNA Damage in EHS/HMS-Induced Cell Death

Given the evidence that EHS and HMS exposure triggers mitochondrial and DNA damage, including elevated ROS levels, loss of mitochondrial membrane potential, increased γ-H2AX expression, and altered expression of mitochondria-related genes, we next carried out rescue experiments to see whether these events are functionally linked to the observed cell death.

**Figure 7:**
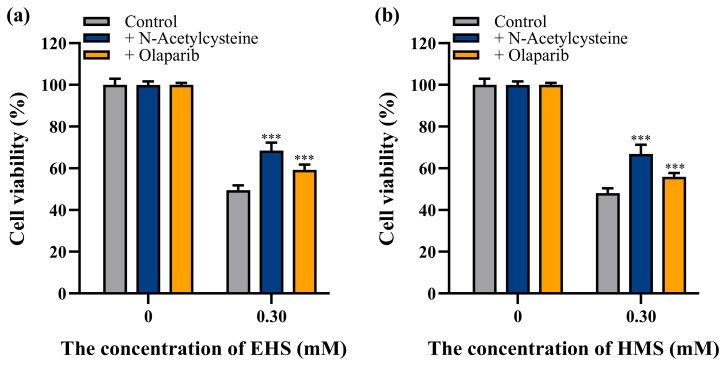
Effect of NAC and Olaparib on cell viability under EHS or HMS exposure. Cells were pretreated with N-acetylcysteine (NAC, 5 mM) or olaparib (10 μM) for 2 h, then co-exposed to 0.30 mM EHS (**a**) or HMS (**b**) for 36 h. Viability was assessed by CCK-8 assay. Data are mean ± SD (*n* = 3). One-way ANOVA followed by Tukey’s test. *** *p* < 0.001 compared with the control group.

To test whether oxidative stress-mediated mitochondrial damage directly contributes to cytotoxicity, we pretreated cells with the antioxidant N-acetylcysteine (NAC). As shown in Figure 7, treatment with 0.30 mM EHS or HMS alone reduced cell viability to 49.5% and 48.0% of control levels, respectively. Notably, NAC (5 mM) pretreatment significantly reversed this effect, bringing viability back up to 68.4% in the EHS-treated group and 66.8% in the HMS-treated group.

The earlier DNA damage results showed increased γ-H2AX expression (Figure 4) and upregulation of *PARP1* by RT-qPCR, both indicative of double-strand breaks. To determine whether DNA damage itself contributes to cell death, we pretreated cells with the PARP inhibitor Olaparib (10 μM) to limit excessive PARP activation during DNA repair. As shown in Figure 7, Olaparib pretreatment significantly attenuated the loss of viability caused by EHS and HMS, raising survival from 49.5% and 48.0% to 59.2% and 55.9%, respectively.

### 2.8. In Vivo Evaluation in Zebrafish

**Figure 8:**
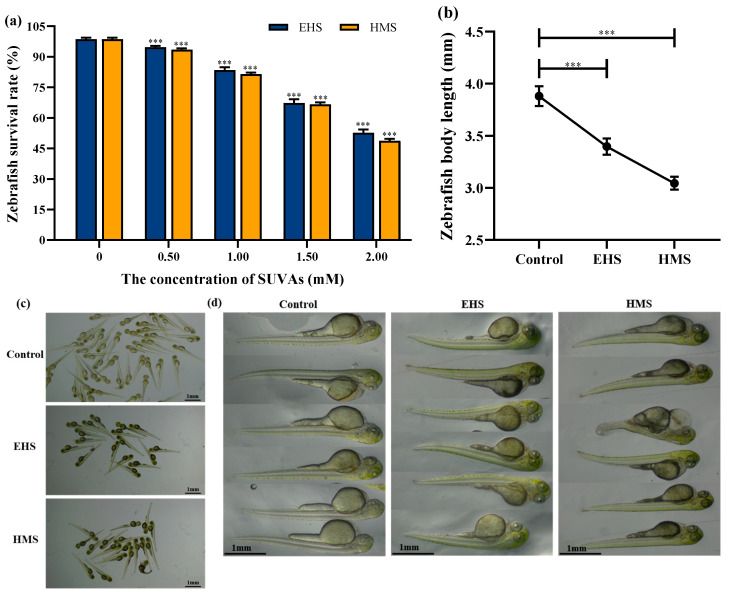

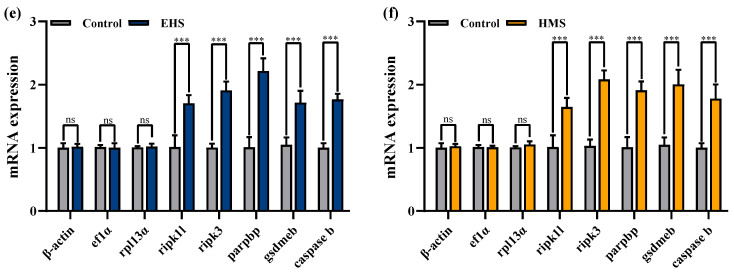
In vivo evaluation in zebrafish. Embryos were exposed to 1 mM EHS or HMS until 72 hpf. (**a**) Survival rate. (**b**) Body length. (**c**,**d**) Developmental toxicity phenotypes. (**e**,**f**) Expression of key genes. Data are mean ± SD (*n* = 3). For normally distributed data, one-way ANOVA followed by Dunnett’s test was used; for non-normally distributed data, the Kruskal–Wallis test was applied. *** *p* < 0.001 compared with the control group.

We next turned to a zebrafish embryo model to see whether the toxic effects observed in our cellular assays could be reproduced in a whole-organism setting. Figure 8a,b show that exposing zebrafish embryos to 1 mM EHS or HMS from 6 hpf to 72 hpf results in a significant decrease in survival rate and a noticeable reduction in body length. Apart from these general impacts, both compounds also led to a variety of developmental abnormalities (Figure 8c,d). In comparison to untreated controls, embryos in the EHS and HMS groups had different degrees of spinal curvature, and most showed signs of cardiac congestion and pericardial fluid buildup. HMS appeared to bring about more serious morphological defects compared to EHS, in line with its greater effects in the in vitro tests. In combination, these observations suggest that both EHS and HMS bring about clear developmental toxicity in zebrafish.

To further check whether the toxic effects seen in vitro occurred in vivo, total RNA was extracted from zebrafish tissues and put through RT-qPCR analysis. The outcomes in Figure 8e,f show that exposure to EHS or HMS significantly upregulated the mRNA expression of genes related to cell death signaling, namely *ripk1l*, *ripk3*, *parpbp*, *gsdmeb*, and *caspase b*. Importantly, the in vivo gene expression changes were highly similar to the molecular alterations detected in earlier in vitro cellular experiments.

## 3. Discussion

The large-scale usage of sunscreen products has resulted in direct and indirect release of organic ultraviolet (UV) filters into marine and other aquatic environments [18], and raised concerns regarding their potential impacts on aquatic organisms [19,55,56,57]. Meanwhile, the frequent presence of these compounds in human urine, serum, and breast milk has raised concerns about the potential impacts on human health [58,59]. Due to their potential ecological and health impacts as new emerging environmental contaminants, organic UV absorbers have attracted increasing attention. In the present study, using a cell model and zebrafish embryo model, we explored the new mechanism of toxicology of salicylate-based sunscreens and provided a theoretical basis for the regulatory control of these compounds.

Our in vitro results showed that EHS and HMS caused a significant, concentration- and time-dependent decrease in cell viability and suppressed proliferation. These effects were accompanied by oxidative stress, as shown by elevated intracellular ROS, altered MDA levels (Figure A1) and SOD activity (Figure A2), and increased LDH release. Concurrently, we observed disrupted calcium homeostasis (elevated Ca^2+^), mitochondrial dysfunction (reduced mitochondrial membrane potential, decreased ATP production), and markedly increased mitochondrial superoxide detected with MitoSOX™ Red. These findings suggest that EHS and HMS severely disturb redox balance, impair mitochondrial function, and compromise membrane integrity. And the observed mitochondrial superoxide accumulation can further damage mitochondria, creating a vicious cycle that amplifies oxidative injury [60,61,62].

We next examined DNA damage and apoptosis pathways. Gene expression analysis revealed upregulation of PARP1 (Figure 5a,b), consistent with the DNA damage detected by γ-H2AX staining, and upregulation of *PKC-α*, a gene involved in calcium signaling, in line with the disturbed Ca^2+^ homeostasis [63,64]. In contrast, key genes downstream of the canonical mitochondrial apoptosis pathway were not significantly changed (Figure A3). To exclude apoptosis more definitively, we performed Western blotting for cleaved caspase-3. As shown in Figure 6h,i, cleaved caspase-3 was not detected in either the control or the treated groups, suggesting that classical mitochondrial apoptosis was not activated. This might be attributable to severe ATP depletion resulting from mitochondrial damage and calcium dysregulation, which limits apoptosome formation and subsequent caspase activation [65,66,67,68].

Importantly, NAC and Olaparib partially rescued cell viability, consistent with the involvement of oxidative stress and DNA damage in the observed cytotoxicity. The strong protective effect of NAC points to oxidative stress as a key driver, while the partial rescue by Olaparib suggests that DNA damage contributes independently. The fact that neither treatment fully prevented cell death suggests the participation of additional mechanisms.

Mitochondrial dysfunction can initiate cell death through multiple pathways. Given the elevated LDH release, indicative of membrane damage, we examined key factors associated with forms of regulated cell death linked to membrane disruption. We observed a significant upregulation of pyroptosis-related genes (*GSDMD*, *Caspase-11*) and necroptosis-related genes (*RIPK1*, *RIPK3*, *ZBP1*, *Caspase-8*). To verify pathway activation at the protein level, we performed further immunoblotting. As shown in Figure 6g–i, the cleaved N-terminal fragment of GSDMD (GSDMD-N) and phosphorylated MLKL (p-MLKL) were strongly detected in EHS- and HMS-treated cells, whereas they were absent in untreated controls. By contrast, cleaved caspase-3 was not detected across all groups. However, several key functional hallmarks remain unaddressed: for pyroptosis, we did not measure IL-1β/IL-18 release, provide direct evidence of membrane pore formation, or assess inflammasome activation; for necroptosis, we did not employ pathway-specific inhibitors (e.g., necrostatin-1) or genetically validate RIPK3/MLKL dependency. Therefore, while these biochemical and transcriptional data are suggestive of pyroptosis and necroptosis involvement, further functional validation, including quantification of secreted IL-1β/IL-18, pore-formation assays, inflammasome characterization, and genetic or pharmacological loss-of-function approaches, will be required in future studies to definitively confirm pathway activation and establish causality.

According to literature reports, Caspase-11 can directly cleave GSDMD to initiate pyroptosis [69], whereas the ZBP1–RIPK3 interaction promotes necroptosis through *Caspase-8* activation [70]; concurrent *PARP1* overactivation may also contribute to parthanatos [71]. Under homeostatic conditions, Caspase-8 cleaves RIPK1 and RIPK3, thereby suppressing necroptosis [72]. However, the high *Caspase-8* expression detected here likely represents an inactive zymogen form, allowing necroptosis to proceed. Notably, when apoptosis or necroptosis is blocked, *Caspase-8* can also mediate pyroptosis, indicating functional plasticity among these pathways [72].

Taken together, our integrated gene expression and protein-level data suggest that EHS and HMS activate pyroptosis and necroptosis via *Caspase-11/GSDMD* and *ZBP1/RIPK3/Caspase-8* axes, respectively, while classical mitochondrial apoptosis is not engaged. The sequence of upstream cellular stress events (oxidative stress, Ca^2+^ imbalance, mitochondrial damage, DNA damage) and subsequent upregulation of key pathway molecules is consistent with established descriptions of these cell death modalities [69,73,74,75,76,77]. However, causal ordering among these upstream events has not been fully resolved in this study. It is also important to note that the relatively high concentrations used here were deliberately selected to robustly and rapidly induce cell death pathway activation for mechanistic dissection. These levels do not reflect environmentally realistic exposure scenarios, and we cannot exclude the possibility that such supra-physiological concentrations activate non-physiological compensatory pathways. Therefore, extrapolation of these findings to environmentally relevant conditions should be undertaken with caution. Whether chronic, low-dose exposure at environmentally relevant concentrations engages the same pathways remains to be investigated in future studies.

Our in vivo zebrafish experiments provided phenotypic and molecular correlates of the in vitro findings. EHS or HMS treatment induced marked developmental toxicity, evidenced by reduced survival and body length, as well as spinal curvature, cardiac congestion, and pericardial edema; HMS consistently exerted greater toxicity. Vertebrate embryogenesis is a highly ordered process of cell proliferation, differentiation, and programmed cell death [78]. If this sequence is disrupted during sensitive developmental windows, resulting cellular or tissue defects may be irreversible and potentially persist to adulthood, causing permanent malformations that compromise individual fitness or population viability [79]. The anomalies we observed, particularly cardiac congestion, pericardial edema, and spinal curvature, suggest that cardiovascular function and body axis formation were affected, which are both essential for early survival [80,81,82]. Similar conclusions have been reached by other groups. For instance, Zhao et al. reported that EHS induced pericardial edema, cardiovascular dysplasia, and ischemia in zebrafish embryos, and considered cardiac defects to be a primary cause of embryonic death [80]. Lu et al. observed developmental defects, reduced heart rate, and significant mortality in EHS-exposed zebrafish, accompanied by transcriptomic disruptions in immunity, lipid metabolism, and oxidative stress pathways [81]. Moreover, Xie et al. demonstrated that parental EHS exposure caused melanin synthesis defects that were transmitted to progeny, suggesting potential transgenerational effects [82].

Consistent with our in vitro observations, genes associated with necroptosis (*ripk1l*, *ripk3*) and pyroptosis (*gsdmeb*, *caspase b*) were also upregulated in zebrafish embryos, suggesting that analogous regulated cell death pathways are engaged in vivo. Indeed, zebrafish *gsdmeb* has been identified as a functional counterpart of mammalian *GSDMD*, capable of inducing neutrophil pyroptosis during bacterial infection [83], and *RIPK3*-dependent necroptosis has been implicated in developmental and inflammatory cell death in zebrafish embryos [84]. The coordinated upregulation of both pyroptotic and necroptotic markers in our study points to possible pathway cooperation, which has also been proposed in other chemically induced toxicity models [85]; however, this possibility awaits direct functional validation. Nevertheless, the consistency across models provides support for the translational relevance of our results and the possibility that both compounds can induce adverse in vivo effects.

It should be noted that the zebrafish in vivo experiments in this study remain at the level of phenotypic and gene expression correlations. Although NAC and Olaparib partially rescued cell viability in vitro, preliminarily implicating oxidative stress and DNA damage in the toxicity, these rescue experiments were not extended to downstream endpoints (e.g., pyroptosis/necroptosis markers) or to the in vivo zebrafish model. Therefore, they serve as upstream functional support for the involvement of oxidative stress and DNA damage, rather than direct evidence that the downstream cell death pathways are causally responsible for the observed toxicity. Likewise, analogous in vivo rescue experiments have not yet been performed to directly confirm causality in the zebrafish model. Consequently, the zebrafish data presented in this study provide evidence supporting the consistency between in vivo toxicity phenotypes and in vitro mechanistic findings, rather than a complete direct verification of the mechanisms. The observed concordance in molecular expression trends across species supports the cross-species conservation of these responses but does not establish causality. Future studies incorporating pharmacological pretreatment, detection of downstream pyroptotic/necroptotic markers, and pathway-specific interventions in zebrafish will be required to establish the causal role of these pathways in vivo.

In conclusion, we provide converging evidence that EHS and HMS induce cell death through pyroptotic and necroptotic pathways, while classical apoptosis is not activated. The parallel gene expression changes observed in zebrafish support the translational relevance of these findings and suggest that these widely used UV filters possess significant biological toxicity. Our results offer a foundation for more comprehensive health risk assessment, informed regulatory decision-making, and the development of safer, more sustainable alternatives. Furthermore, we support a model in which EHS and HMS trigger oxidative stress, calcium dysregulation, mitochondrial dysfunction, and DNA damage, ultimately leading to pyroptotic and necroptotic cell death. Among these steps, elevated ROS levels, disrupted calcium homeostasis, mitochondrial dysfunction, and DNA damage have all been experimentally confirmed in this study. However, the causal link between ROS and Ca^2+^ dysregulation is a reasonable inference drawn from existing literature; the precise sequence from Ca^2+^ overload to mitochondrial damage has not been definitively established in the present work, and it remains to be determined whether mitochondrial damage is the sole upstream trigger of DNA damage or whether these events occur in parallel. Future research needs to use step-specific inhibitors and genetic interventions to further clarify these relationships.

## 4. Materials and Methods

### 4.1. Reagents and Equipment

Mouse embryonic fibroblast 3T6 cells were obtained from Suzhou Haixing Biosciences Co., Ltd. (Suzhou, China) (https://www.cas9x.com/). EHS (≥98%) and HMS (≥98%) were sourced from Shanghai Aladdin Biochemical Technology Co., Ltd. (Shanghai, China).

### 4.2. Solution Preparation

Stock solutions of EHS and HMS (1 M) were dissolved in DMSO and kept at −20 °C away from light. Working solutions (0.05, 0.1, 0.3, 0.5, 0.8, 1.0 mM) were obtained by diluting the stock in complete medium, with DMSO not exceeding 0.1%, and held at 4 °C in the dark.

### 4.3. Cell Viability Assay(CCK8)

3T6 cells (1 × 10^4^/well) seeded in 96-well plates were allowed to adhere for 24 h before 24 or 36 h exposure to EHS or HMS (0.05, 0.1, 0.3, 0.5, 0.8, 1.0 mM). The supernatant was then replaced with CCK-8-containing medium (10%), and absorbance at 410 nm was measured after 2 h on a Cytation 5 reader (BioTek, Winooski, VT, USA), using the untreated group containing 0.1% DMSO as the baseline [86].

### 4.4. EdU-488 Cell Proliferation Assay

An EdU-488 incorporation kit was used to quantify newly synthesized DNA via click-chemistry fluorescent labeling. Cells (2 × 10^4^/well) seeded in 48-well plates were allowed to attach for 24 h before treatment with EHS or HMS (0.1, 0.3, or 0.5 mM) for 36 h. The EdU working solution was then introduced for 30 min in the dark, followed by Hoechst 33342 for 10 min. Fluorescence was captured with an Axio Observer microplate reader (ZEISS, Oberkochen, Germany).

### 4.5. Calcein/PI Cell Viability and Cytotoxicity Assays

Cell survival and death were distinguished via Calcein-AM/PI dual staining; following exposure, cells were labeled for 30 min at 37 °C in the dark and examined under an Axio Observer fluorescence microscope (ZEISS, Oberkochen, Germany).

### 4.6. Annexin V-FITC/PI Double Staining to Detect Apoptosis

Using an Annexin V-FITC/PI kit, collected cells were PBS-washed, dual-stained for 15 min in the dark, and apoptosis rates were measured by flow cytometry (Canto II, BD, San Jose, CA, USA) [87].

### 4.7. Oxidative Stress System Detection

Oxidative stress markers were measured as follows. Cells were incubated with the DCFH-DA probe at 37 °C in the dark for 20 min, and fluorescence was measured using a multifunctional microplate reader (Axio Observer, ZEISS, Oberkochen, Germany). The assays for superoxide dismutase (SOD) activity and malondialdehyde (MDA) levels were performed according to Tian et al. [23].

### 4.8. Intracellular Ca^2+^ Content Detection

Intracellular Ca^2+^ levels were measured using the Fluo-4 AM calcium fluorescent probe. Briefly, cells were incubated with Fluo-4 AM at 37 °C for 30 min, then fluorescence was measured to determine changes in intracellular Ca^2+^ levels [88].

### 4.9. Mitochondrial Membrane Potential (MMP) JC-1 Detection

To evaluate mitochondrial membrane potential, cells were treated with JC-1 working solution (0.5 mL/well) for 20 min in the dark, washed twice with staining buffer, and fluorescence was detected via an Axio Observer microplate reader (ZEISS, Oberkochen, Germany) [23].

### 4.10. ATP Content Detection

ATP levels were measured using an ATP assay kit to evaluate the impact of mitochondrial dysfunction on cellular energy status. Briefly, the ATP detection working solution was incubated for 3–5 min, then an equal volume of cell lysate supernatant was added and mixed. Luminescence was measured using a microplate reader in luminescence mode (Cytation5, Biotek, Winooski, VT, USA) [89].

### 4.11. Mitochondrial Superoxide Detection

MitoSOX Red kit (Beyotime, Shanghai, China) was applied to assess mitochondrial superoxide levels. Following treatment, cells were stained for 30 min in the dark, rinsed with PBS, and imaged using a laser confocal microscope (TCS SP8, Leica, Wetzlar, Germany) [48].

### 4.12. LDH Enzyme Activity Detection

Cell membrane integrity was evaluated by quantifying LDH release from collected supernatants. After adding LDH substrate solution (100 µL/sample) and incubating at 37 °C for 30 min, absorbance at 450 nm was recorded with a microplate reader.

### 4.13. DNA Damage Immunofluorescence Detection

DNA damage was assessed by γ-H2AX immunofluorescence. 3T6 cells were seeded in 24-well plates and treated as described above. According to the manufacturer’s instructions, cells were stained using a γ-H2AX DNA damage detection kit to evaluate DNA damage after EHS or HMS exposure. Fluorescence was measured using a multifunctional microplate reader (Axio Observer, ZEISS, Oberkochen, Germany) [90].

### 4.14. Quantitative Real-Time PCR (qRT-PCR)

RNA extracted from 3T6 cells and zebrafish embryos via an Animal Total RNA Extraction Kit was converted to cDNA and amplified by SYBR Green-based qRT-PCR (QuantStudio 6 Flex, Thermo Fisher, Waltham, MA, USA; 95 °C/2 min, 40 cycles of 95 °C/15 s and 60 °C/30 s). GAPDH-normalized mRNA abundance was determined using the 2^−△△Ct^ method.

### 4.15. Western Blot

Cell lysates were centrifuged (12,000 rpm, 4 °C, 20 min), and supernatants were harvested for protein quantification. Equal protein amounts were separated by 12% SDS-PAGE, blotted onto PVDF membranes, and blocked with 5% skim milk/TBST for 1 h. Membranes were incubated overnight at 4 °C with primary antibodies, washed, and then probed with HRP-linked secondary antibodies for 1 h. Signals were developed by ECL, and band intensities were quantified using ImageJ (v1.54p).

### 4.16. Rescue Experiment

To check the roles of mitochondrial and DNA damage in cytotoxicity induced by EHS/HMS, a rescue experiment was done with the CCK-8 assay. Log-phase 3T6 cells were seeded in 96-well plates at a density of 5 × 10^3^ cells/well and cultured overnight. Cells were then pretreated for 2 h with 5 mM N-acetylcysteine or 10 μM PARP inhibitor Olaparib individually, followed by co-treatment with EHS (0.30 mM) or HMS (0.30 mM) for 36 h. Fresh medium with 10% CCK-8 was added after removing the original supernatant, incubated at 37 °C for 2 h, and the absorbance at 410 nm was recorded. Viability was normalized to the control group, and all assays were performed in triplicate across three independent experiments.

### 4.17. Zebrafish In Vivo Toxicity Assessment

Wild-type AB zebrafish (*Danio rerio*; 5 months old; male: female = 1:1) were used for embryo collection. Adult fish were placed in breeding tanks with a divider overnight, and spawning was induced by light onset the following morning. Fertilized eggs were collected within 2 h post-fertilization (hpf). Viable embryos were selected under a stereomicroscope and maintained in E3 embryo medium containing 5 mM NaCl, 0.17 mM KCl, 0.33 mM MgSO_4_, 0.33 mM CaCl_2_, and 0.0001% methylene blue at 28 °C.

At 6 hpf, embryos were randomly assigned to the control group (E3 medium only) or the exposure groups. The test concentration was determined based on a preliminary experiment, and 1 mM was selected for subsequent exposure. Each treatment consisted of three replicates, with 15 embryos per replicate. Embryos were continuously exposed until 72 hpf under a 14 h light/10 h dark photoperiod, and the exposure solutions were renewed every 24 h.

Embryo survival and developmental abnormalities were evaluated daily throughout the exposure period. Dead embryos were removed promptly and were identified according to standard morphological criteria, including coagulation, absence of heartbeat, and non-detachment of the tail from the yolk sac. Mortality and malformation rates were recorded for each group.

Hatching was monitored at 12 h intervals beginning at 48 hpf. Morphological assessments were performed at 24, 48, and 72 hpf under a stereomicroscope, with particular attention to toxicological endpoints including pericardial edema and spinal curvature. All observations were conducted in a blinded manner. Body length was measured at 72 hpf as an indicator of larval growth. The exposure experiment was independently repeated three times.

For molecular analysis, larvae were collected at 72 hpf. Five larvae were pooled to generate one sample, and three biological replicates were prepared for each group. Total RNA was extracted using TRIzol reagent, followed by reverse transcription to synthesize cDNA. The mRNA expression levels of apoptosis-related genes were quantified by RT-qPCR using β-actin as the reference gene.

### 4.18. Statistical Analysis

All experiments were performed in three biological replicates, and the results are presented as mean ± standard deviation (SD). ImageJ software was used to quantify fluorescence intensity, and FlowJo (v10.8.1), Origin 2024, and GraphPad Prism (v10.1.2) were used for data analysis and graphing. Statistical comparisons were made using one-way ANOVA or Student’s *t*-test, the normality of the concentration data for each substance was tested using a one-sample K-S test, and comparisons between two groups were made using an independent sample *t*-test. *p* < 0.05 was considered statistically significant.

## 5. Conclusions

In summary, this study proposes a molecular cascade through which the widely used salicylate-type UV absorbers EHS and HMS induce cytotoxicity. In vitro, exposure to these compounds triggered oxidative stress, disrupted Ca^2+^ homeostasis, caused mitochondrial dysfunction, and induced DNA damage. These cellular injuries collectively activated two lytic programmed cell death pathways, *RIPK1/RIPK3*-mediated necroptosis and *Caspase-11*-dependent pyroptosis, as suggested by increased phosphorylation of MLKL and cleavage of GSDMD, respectively. In contrast, classical mitochondrial apoptosis was not engaged, consistent with the absence of cleaved Caspase-3. While the individual upstream stress events were thus experimentally verified, the causal links connecting them (e.g., ROS to Ca^2+^ dysregulation to mitochondrial damage to DNA damage) remain largely inferred, and their hierarchical ordering has not been resolved.

Consistent with the in vitro findings, EHS and HMS exposure in zebrafish embryos induced pronounced developmental toxicity, including reduced survival, shortened body length, and morphological abnormalities. The upregulation of cell death-related genes (*ripk1l*, *ripk3*, *parpbp*, *gsdmeb*, *caspase b*) mirrored the molecular signatures observed in vitro, providing in vivo phenotypic and transcriptional correlates. However, in the absence of targeted rescue experiments or pathway-specific interventions in vivo, these data only provide supporting evidence, rather than establishing causality.

Several limitations should be noted. The concentrations used in this study were selected to robustly induce cell death pathways for mechanistic dissection and are substantially higher than environmentally relevant exposure levels; extrapolation to realistic scenarios requires caution. Furthermore, although pyroptosis and necroptosis are supported by protein-level markers (GSDMD-N, p-MLKL), functional hallmarks such as IL-1β/IL-18 release, membrane pore formation, and inhibitor-based rescue remain to be addressed. Therefore, the present work defines a mechanistic framework linking upstream stress signals to regulated cell death programs, but definitive causal relationships await future studies employing step-specific inhibitors, genetic loss-of-function approaches, and chronic low-dose exposure models.

Overall, by delineating the molecular events from initial cellular stress to cell death execution, this study provides a mechanistic basis for evidence-based safety assessments and the development of safer, more sustainable sunscreen products, contributing to the long-term goals of protecting both human health and ecological integrity.

## Data Availability

The original contributions presented in this study are included in the article. Further inquiries can be directed to the corresponding authors.

## Abbreviations

The following abbreviations are used in this manuscript:

OUVAs	Organic ultraviolet absorbers
EHS	2-ethylhexyl salicylates
HMS	Homosalate
ROS	Reactive oxygen species

## References

[1] Rajakumar K., Greenspan S.L., Thomas S.B., Holick M.F. (2007). SOLAR Ultraviolet Radiation AND Vitamin D: A Historical Perspective. Am. J. Public Health.

[2] Adil M.D., Kaiser P., Satti N.K., Zargar A.M., Vishwakarma R.A., Tasduq S.A. (2010). Effect of Emblica Officinalis (Fruit) against UVB-Induced Photo-Aging in Human Skin Fibroblasts. J. Ethnopharmacol..

[3] Chaiyabutr C., Sukakul T., Kumpangsin T., Bunyavaree M., Charoenpipatsin N., Wongdama S., Boonchai W. (2021). Ultraviolet Filters in Sunscreens and Cosmetic Products—A Market Survey. Contact Dermat..

[4] Jesus A., Sousa E., Cruz M.T., Cidade H., Lobo J.M.S., Almeida I.F. (2022). UV Filters: Challenges and Prospects. Pharmaceuticals.

[5] Zhang Z., Ren N., Li Y.-F., Kunisue T., Gao D., Kannan K. (2011). Determination of Benzotriazole and Benzophenone UV Filters in Sediment and Sewage Sludge. Environ. Sci. Technol..

[6] Gautam K., Anbumani S. (2024). Understudied and Underestimated Impacts of Organic UV Filters on Terrestrial Ecosystems. Sci. Total Environ..

[7] Tsui M.M.P., Leung H.W., Wai T.-C., Yamashita N., Taniyasu S., Liu W., Lam P.K.S., Murphy M.B. (2014). Occurrence, Distribution and Ecological Risk Assessment of Multiple Classes of UV Filters in Surface Waters from Different Countries. Water Res..

[8] Langford K.H., Reid M.J., Fjeld E., Øxnevad S., Thomas K.V. (2015). Environmental Occurrence and Risk of Organic UV Filters and Stabilizers in Multiple Matrices in Norway. Environ. Int..

[9] Tsui M.M.P., Chen L., He T., Wang Q., Hu C., Lam J.C.W., Lam P.K.S. (2019). Organic Ultraviolet (UV) Filters in the South China Sea Coastal Region: Environmental Occurrence, Toxicological Effects and Risk Assessment. Ecotoxicol. Environ. Saf..

[10] Mao J.F., Li W., Ong C.N., He Y., Jong M.-C., Gin K.Y.-H. (2022). Assessment of Human Exposure to Benzophenone-Type UV Filters: A Review. Environ. Int..

[11] Vuckovic D., Tinoco A.I., Ling L., Renicke C., Pringle J.R., Mitch W.A. (2022). Conversion of Oxybenzone Sunscreen to Phototoxic Glucoside Conjugates by Sea Anemones and Corals. Science.

[12] Joensen U.N., Jørgensen N., Thyssen J.P., Szecsi P.B., Stender S., Petersen J.H., Andersson A.-M., Frederiksen H. (2018). Urinary Excretion of Phenols, Parabens and Benzophenones in Young Men: Associations to Reproductive Hormones and Semen Quality Are Modified by Mutations in the Filaggrin Gene. Environ. Int..

[13] Hines E.P., Mendola P., Von Ehrenstein O.S., Ye X., Calafat A.M., Fenton S.E. (2015). Concentrations of Environmental Phenols and Parabens in Milk, Urine and Serum of Lactating North Carolina Women. Reprod. Toxicol..

[14] Lu H., Wang Z., Cui H.B., Jin Y.H., Yang F., Feng L.L., Hu X.F., Shen Z.M., Yuan T. (2021). Identifications and characteristics of organic ultraviolet filters in indoor air. J. Environ. Occup. Med..

[15] Picone M., Del Vecchio S., Vecchiato M., Tagliapietra A., Gambaro A., Volpi Ghirardini A. (2025). The UV Filters Ethyl-Hexyl Salicylate and Octocrylene Affects Feeding and Reproduction in the Marine Copepod Acartia Tonsa. Mar. Environ. Res..

[16] Bury D., Weber T., Ebert K.E., Zülz S., Brüning T., Koch H.M., Kolossa-Gehring M. (2023). Increasing Exposure to the UV Filters Octocrylene and 2-Ethylhexyl Salicylate in Germany from 1996 to 2020: Human Biomonitoring in 24-h Urine Samples of the German Environmental Specimen Bank (ESB). Environ. Int..

[17] Ebert K.E., Belov V.N., John M., Weiss T., Brüning T., Hayen H., Koch H.M., Bury D. (2024). Identification, Organic Synthesis, and Sensitive Analysis of a *Cis* -Homosalate-Specific Exposure Biomarker. Chem. Res. Toxicol..

[18] Huang Y., Law J.C.-F., Lam T.-K., Leung K.S.-Y. (2021). Risks of Organic UV Filters: A Review of Environmental and Human Health Concern Studies. Sci. Total Environ..

[19] Cadena-Aizaga M.I., Montesdeoca-Esponda S., Torres-Padrón M.E., Sosa-Ferrera Z., Santana-Rodríguez J.J. (2020). Organic UV Filters in Marine Environments: An Update of Analytical Methodologies, Occurrence and Distribution. Trends Environ. Anal. Chem..

[20] Danovaro R., Corinaldesi C. (2003). Sunscreen Products Increase Virus Production through Prophage Induction in Marine Bacterioplankton. Microb. Ecol..

[21] Paredes E., Perez S., Rodil R., Quintana J.B., Beiras R. (2014). Ecotoxicological Evaluation of Four UV Filters Using Marine Organisms from Different Trophic Levels Isochrysis Galbana, Mytilus Galloprovincialis, Paracentrotus Lividus, and Siriella Armata. Chemosphere.

[22] Yang C., Lim W., Bazer F.W., Song G. (2018). Homosalate Aggravates the Invasion of Human Trophoblast Cells as Well as Regulates Intracellular Signaling Pathways Including PI3K/AKT and MAPK Pathways. Environ. Pollut..

[23] Tian L., Guo M., Chen H., Wu Y. (2023). Human Health Risk Assessment of Cinnamate UV Absorbers: In Vitro and in Silico Investigations. Environ. Int..

[24] Ye R., Li Z., Xian H., Zhong Y., Liang B., Huang Y., Chen D., Dai M., Tang S., Guo J. (2024). Combined Effects of Polystyrene Nanosphere and Homosolate Exposures on Estrogenic End Points in MCF-7 Cells and Zebrafish. Environ. Health Perspect..

[25] Těšínská P., Škarohlíd R., Kroužek J., McGachy L. (2024). Environmental Fate of Organic UV Filters: Global Occurrence, Transformation, and Mitigation via Advanced Oxidation Processes. Environ. Pollut..

[26] Couceiro B., Hameed H., Vieira A.C.F., Singh S.K., Dua K., Veiga F., Pires P.C., Ferreira L., Paiva-Santos A.C. (2025). Promoting Health and Sustainability: Exploring Safer Alternatives in Cosmetics and Regulatory Perspectives. Sustain. Chem. Pharm..

[27] Chemicals and Waste Management: Essential to Achieving the Sustainable Development Goals. https://iomc.info/publications/m/item/iomc-brochure-on-sdg.

[28] Azevedo R.D.S., Falcão K.V.G., Assis C.R.D., Martins R.M.G., Araújo M.C., Yogui G.T., Neves J.L., Seabra G.M., Maia M.B.S., Amaral I.P.G. (2021). Effects of Pyriproxyfen on Zebrafish Brain Mitochondria and Acetylcholinesterase. Chemosphere.

[29] Yang L., Huang T., Li R., Souders C.L., Rheingold S., Tischuk C., Li N., Zhou B., Martyniuk C.J. (2021). Evaluation and Comparison of the Mitochondrial and Developmental Toxicity of Three Strobilurins in Zebrafish Embryo/Larvae. Environ. Pollut..

[30] Bai C., Zheng Y., Tian L., Lin J., Song Y., Huang C., Dong Q., Chen J. (2023). Structure-Based Developmental Toxicity and ASD-Phenotypes of Bisphenol A Analogues in Embryonic Zebrafish. Ecotoxicol. Environ. Saf..

[31] Xiang P., Jia Y., Wang K., Li M.-Y., Qin Y.-S., He R.-W., Gao P., Liu Y., Liu X., Ma L.Q. (2018). Water Extract of Indoor Dust Induces Tight Junction Disruption in Normal Human Corneal Epithelial Cells. Environ. Pollut..

[32] (2015). Safety and Technical Standards for Cosmetics. State Food and Drug Administration, Announcement No. 268. https://www.nmpa.gov.cn/hzhp/hzhpfgwj/hzhpgzwj/20151223120001986.html?type=pc&m=.

[33] Simon H.U., Yehia A.H., Schaffer F.L. (2001). Role of Reactive Oxygen Species (ROS) in Apoptosis Induction. Apoptosis.

[34] Bong A.H.L., Monteith G.R. (2018). Calcium Signaling and the Therapeutic Targeting of Cancer Cells. Biochim. Biophys. Acta BBA Mol. Cell Res..

[35] Parkash J., Asotra K. (2010). Calcium Wave Signaling in Cancer Cells. Life Sci..

[36] Yang M., Hu Y., Hao X., Chen Q., Cao Y., Ran H., Zhang W. (2025). Ultrasound-Actuated Ion Homeostasis Perturbator for Oxidative Damage-Augmented Ca^2+^ Interference Therapy and Combined Immunotherapy. Mater. Today Bio.

[37] Yan Y., Wei C.-L., Zhang W.-R., Cheng H.-P., Liu J. (2006). Cross-Talk between Calcium and Reactive Oxygen Species. Acta Pharmacol. Sin..

[38] De Nicolo B., Cataldi-Stagetti E., Diquigiovanni C., Bonora E. (2023). Calcium and Reactive Oxygen Species Signaling Interplays in Cardiac Physiology and Pathologies. Antioxidants.

[39] Huang Y., Prastyaningrum L.L., Wang X., Xu F., Wang Z., Wang Z., Tan X., Dai G., Chen G., Gong X. (2025). MICU1 Is the Nexus for CaV3.3 Regulation of Mitochondrial Calcium, Redox Balance and Chondrocyte Viability. Int. J. Biol. Macromol..

[40] Antonucci S., Di Lisa F., Kaludercic N. (2021). Mitochondrial Reactive Oxygen Species in Physiology and Disease. Cell Calcium.

[41] Vaishampayan P., Lee Y. (2024). Redox-Active Vitamin C Suppresses Human Osteosarcoma Growth by Triggering Intracellular ROS-Iron–Calcium Signaling Crosstalk and Mitochondrial Dysfunction. Redox Biol..

[42] Gottlieb E., Armour S.M., Harris M.H., Thompson C.B. (2003). Mitochondrial Membrane Potential Regulates Matrix Configuration and Cytochrome c Release during Apoptosis. Cell Death Differ..

[43] Sivandzade F., Bhalerao A., Cucullo L. (2019). Analysis of the Mitochondrial Membrane Potential Using the Cationic JC-1 Dye as a Sensitive Fluorescent Probe. Bio-Protocol.

[44] Zorova L.D., Popkov V.A., Plotnikov E.J., Silachev D.N., Pevzner I.B., Jankauskas S.S., Zorov S.D., Babenko V.A., Zorov D.B. (2018). Functional Significance of the Mitochondrial Membrane Potential. Biochem. Mosc. Suppl. A Membr. Cell Biol..

[45] Mattiasson G. (2004). Analysis of Mitochondrial Generation and Release of Reactive Oxygen Species. Cytom. Pt. A.

[46] Mathieu B., Rondeau J.D., Mignion L., Sonveaux P., Gallez B. (2025). Noninvasive in Vivo Discrimination between Mitochondrial ROS and Global ROS Production in Solid Tumors Using EPR Spectroscopy. Redox Biol..

[47] Mailloux R.J. (2021). An Update on Methods and Approaches for Interrogating Mitochondrial Reactive Oxygen Species Production. Redox Biol..

[48] Johnson-Cadwell L.I., Jekabsons M.B., Wang A., Polster B.M., Nicholls D.G. (2007). ‘Mild Uncoupling’ Does Not Decrease Mitochondrial Superoxide Levels in Cultured Cerebellar Granule Neurons but Decreases Spare Respiratory Capacity and Increases Toxicity to Glutamate and Oxidative Stress. J. Neurochem..

[49] Liu B.-H., Yu F.-Y., Wu T.-S., Li S.-Y., Su M.-C., Wang M.-C., Shih S.-M. (2003). Evaluation of Genotoxic Risk and Oxidative DNA Damage in Mammalian Cells Exposed to Mycotoxins, Patulin and Citrinin. Toxicol. Appl. Pharmacol..

[50] Li Y., Wei L., Cao J., Qiu L., Jiang X., Li P., Song Q., Zhou H., Han Q., Diao X. (2016). Oxidative Stress, DNA Damage and Antioxidant Enzyme Activities in the Pacific White Shrimp (*Litopenaeus vannamei*) When Exposed to Hypoxia and Reoxygenation. Chemosphere.

[51] Xu X., Pang Y., Fan X. (2025). Mitochondria in Oxidative Stress, Inflammation and Aging: From Mechanisms to Therapeutic Advances. Signal Transduct. Target. Ther..

[52] Eldakhakhny B.M., Abdulaal W.H., Khan J., Ajoolabady A., Pratico D., Bahijri S., Tuomilehto J., Borai A., Kim B., Ren J. (2026). Mitochondria in Pyroptosis: Mechanisms and Implications. Mol. Immunol..

[53] Chung J.Y., Knutson B.A. (2025). Bypassing the Guardian: Regulated Cell Death Pathways in P53-Mutant Cancers. Cell. Mol. Biol. Lett..

[54] Markert C.L. (1984). Lactate Dehydrogenase. Biochemistry and Function of Lactate Dehydrogenase. Cell Biochem. Funct..

[55] Ozáez I., Morcillo G., Martínez-Guitarte J.-L. (2016). Ultraviolet Filters Differentially Impact the Expression of Key Endocrine and Stress Genes in Embryos and Larvae of Chironomus Riparius. Sci. Total Environ..

[56] Campos D., Gravato C., Quintaneiro C., Golovko O., Žlábek V., Soares A.M.V.M., Pestana J.L.T. (2017). Toxicity of Organic UV-Filters to the Aquatic Midge Chironomus Riparius. Ecotoxicol. Environ. Saf..

[57] Giraldo A., Montes R., Rodil R., Quintana J.B., Vidal-Liñán L., Beiras R. (2017). Ecotoxicological Evaluation of the UV Filters Ethylhexyl Dimethyl P-Aminobenzoic Acid and Octocrylene Using Marine Organisms Isochrysis Galbana, Mytilus Galloprovincialis and Paracentrotus Lividus. Arch. Environ. Contam. Toxicol..

[58] Ruszkiewicz J.A., Pinkas A., Ferrer B., Peres T.V., Tsatsakis A., Aschner M. (2017). Neurotoxic Effect of Active Ingredients in Sunscreen Products, a Contemporary Review. Toxicol. Rep..

[59] Ahn S., An S., Lee M., Lee E., Pyo J.J., Kim J.H., Ki M.W., Jin S.H., Ha J., Noh M. (2019). A Long-Wave UVA Filter Avobenzone Induces Obesogenic Phenotypes in Normal Human Epidermal Keratinocytes and Mesenchymal Stem Cells. Arch. Toxicol..

[60] Rizwan H., Pal S., Sabnam S., Pal A. (2020). High Glucose Augments ROS Generation Regulates Mitochondrial Dysfunction and Apoptosis via Stress Signalling Cascades in Keratinocytes. Life Sci..

[61] Zhang J., Wang B., Wang H., He H., Wu Q., Qin X., Yang X., Chen L., Xu G., Yuan Z. (2018). Disruption of the Superoxide Anions-Mitophagy Regulation Axis Mediates Copper Oxide Nanoparticles-Induced Vascular Endothelial Cell Death. Free Radic. Biol. Med..

[62] Kim E.H., Choi K.S. (2008). A Critical Role of Superoxide Anion in Selenite-Induced Mitophagic Cell Death. Autophagy.

[63] Chappidi N., Quail T., Doll S., Vogel L.T., Aleksandrov R., Felekyan S., Kühnemuth R., Stoynov S., Seidel C.A.M., Brugués J. (2024). PARP1-DNA Co-Condensation Drives DNA Repair Site Assembly to Prevent Disjunction of Broken DNA Ends. Cell.

[64] Bolander J., Chai Y.C., Geris L., Schrooten J., Lambrechts D., Roberts S.J., Luyten F.P. (2016). Early BMP, Wnt and Ca^2+^/PKC Pathway Activation Predicts the Bone Forming Capacity of Periosteal Cells in Combination with Calcium Phosphates. Biomaterials.

[65] Petersen O.H., Gerasimenko J.V., Gerasimenko O.V., Gryshchenko O., Peng S. (2021). The Roles of Calcium and ATP in the Physiology and Pathology of the Exocrine Pancreas. Physiol. Rev..

[66] Balakrishnan K., Stellrecht C.M., Genini D., Ayres M., Wierda W.G., Keating M.J., Leoni L.M., Gandhi V. (2005). Cell Death of Bioenergetically Compromised and Transcriptionally Challenged CLL Lymphocytes by Chlorinated ATP. Blood.

[67] Hung W.-Y., Chang J.-H., Cheng Y., Cheng G.-Z., Huang H.-C., Hsiao M., Chung C.-L., Lee W.-J., Chien M.-H. (2019). Autophagosome Accumulation-Mediated ATP Energy Deprivation Induced by Penfluridol Triggers Nonapoptotic Cell Death of Lung Cancer via Activating Unfolded Protein Response. Cell Death Dis..

[68] Giorgi C., Marchi S., Pinton P. (2018). The Machineries, Regulation and Cellular Functions of Mitochondrial Calcium. Nat. Rev. Mol. Cell Biol..

[69] Kayagaki N., Stowe I.B., Lee B.L., O’Rourke K., Anderson K., Warming S., Cuellar T., Haley B., Roose-Girma M., Phung Q.T. (2015). Caspase-11 Cleaves Gasdermin D for Non-Canonical Inflammasome Signalling. Nature.

[70] Newton K., Strasser A., Kayagaki N., Dixit V.M. (2024). Cell Death. Cell.

[71] Morris G., Walker A.J., Berk M., Maes M., Puri B.K. (2018). Cell Death Pathways: A Novel Therapeutic Approach for Neuroscientists. Mol. Neurobiol..

[72] Willson J. (2020). A Matter of Life and Death for Caspase 8. Nat. Rev. Mol. Cell Biol..

[73] Mishra S., Jain D., Dey A.A., Nagaraja S., Srivastava M., Khatun O., Balamurugan K., Anand M., Ashok A.K., Tripathi S. (2024). Bat RNA Viruses Employ Viral RHIMs Orchestrating Species-Specific Cell Death Programs Linked to Z-RNA Sensing and ZBP1-RIPK3 Signaling. iScience.

[74] Bao Y., Feng Z., Niu Y., Hu Q., Liu H., Zhang H., Li J. (2025). Esketamine Mitigates Endotoxin-Induced Acute Lung Injury by Suppressing Caspase-11-Driven Pyroptosis. BMC Anesthesiol..

[75] Szczerba M., Johnson B., Acciai F., Gogerty C., McCaughan M., Williams J., Kibler K.V., Jacobs B.L. (2023). Canonical Cellular Stress Granules Are Required for Arsenite-Induced Necroptosis Mediated by Z-DNA–Binding Protein 1. Sci. Signal..

[76] Tye H., Conos S.A., Djajawi T.M., Abdoulkader N., Kong I.Y., Kammoun H.L., Narayana V.K., Speir M., Gottschalk T.A., Emery J. (2023). Divergent Roles for Caspase-8 and MLKL in High-Fat Diet Induced Obesity and NAFLD in Mice. bioRxiv.

[77] Drummer C., Saaoud F., Jhala N.C., Cueto R., Sun Y., Xu K., Shao Y., Lu Y., Shen H., Yang L. (2023). Caspase-11 Promotes High-Fat Diet-Induced NAFLD by Increasing Glycolysis, OXPHOS, and Pyroptosis in Macrophages. Front. Immunol..

[78] Kimmel C.B., Ballard W.W., Kimmel S.R., Ullmann B., Schilling T.F. (1995). Stages of Embryonic Development of the Zebrafish. Dev. Dyn..

[79] Hill A.J., Teraoka H., Heideman W., Peterson R.E. (2005). Zebrafish as a Model Vertebrate for Investigating Chemical Toxicity. Toxicol. Sci..

[80] Zhao G., Gao M., Guo S., Zeng S., Ye C., Wang M., Anwar Z., Hu B., Hong Y. (2023). UV Filter Ethylhexyl Salicylate Affects Cardiovascular Development by Disrupting Lipid Metabolism in Zebrafish Embryos. Sci. Total Environ..

[81] Lu X., Sun L., Chen J., Wang J., Guan M., Xu S. (2025). Comparative Molecular Insights into Developmental and Behavioral Toxicity Induced by Octocrylene and Ethylhexyl Salicylate Exposure on Zebrafish. Integr. Zool..

[82] Xie Z., Zhou R., Ding Z., Zhou D., Jin Q. (2022). Melanin Interference Toxicity or Transgenerational Toxicity of Organic UV Filter Ethylhexyl Salicylate on Zebrafish. Sci. Total Environ..

[83] Lozano-Gil J.M., Rodríguez-Ruiz L., Tyrkalska S.D., García-Moreno D., Pérez-Oliva A.B., Mulero V. (2022). Gasdermin E Mediates Pyroptotic Cell Death of Neutrophils and Macrophages in a Zebrafish Model of Chronic Skin Inflammation. Dev. Comp. Immunol..

[84] Wen W., Chen J., Zhou Y., Li G., Zhang Y. (2022). Loss of Ripk3 Attenuated Neutrophil Accumulation in a Lipopolysaccharide-Induced Zebrafish Inflammatory Model. Cell Death Discov..

[85] Frank D., Vince J.E. (2019). Pyroptosis versus Necroptosis: Similarities, Differences, and Crosstalk. Cell Death Differ..

[86] Tan G., Huang C., Chen J., Zhi F. (2020). HMGB1 Released from GSDME-Mediated Pyroptotic Epithelial Cells Participates in the Tumorigenesis of Colitis-Associated Colorectal Cancer through the ERK1/2 Pathway. J. Hematol. Oncol..

[87] Qiu M., Chen J., Liu M., Nie Z., Ke M., Dong G., Zhao H., Zhou C., Zeng H., He B. (2024). Single-Cell RNA Sequencing Reveals the Role of Mitochondrial Dysfunction in the Cardiogenic Toxicity of Perfluorooctane Sulfonate in Human Embryonic Stem Cells. Ecotoxicol. Environ. Saf..

[88] Yang L., Zhang L., He J., Tu C., Li S., Wang X., Wang L. (2017). Formation and Properties of Tangential Discontinuities in Three-Dimensional Compressive MHD Turbulence. Astrophys. J..

[89] Wang P., Cui Y., Liu Y., Li Z., Bai H., Zhao Y., Chang Y.-Z. (2022). Mitochondrial Ferritin Alleviates Apoptosis by Enhancing Mitochondrial Bioenergetics and Stimulating Glucose Metabolism in Cerebral Ischemia Reperfusion. Redox Biol..

[90] Zhang Q., Chen C., Zou X., Wu W., Di Y., Li N., Fu A. (2024). Iron Promotes Ovarian Cancer Malignancy and Advances Platinum Resistance by Enhancing DNA Repair via FTH1/FTL/POLQ/RAD51 Axis. Cell Death Dis..

